# Characterization of the global profile of genes expressed in cervical epithelium by Serial Analysis of Gene Expression (SAGE)

**DOI:** 10.1186/1471-2164-6-130

**Published:** 2005-09-19

**Authors:** Carlos Pérez-Plasencia, Gregory Riggins, Guelaguetza Vázquez-Ortiz, José Moreno, Hugo Arreola, Alfredo Hidalgo, Patricia Piña-Sanchez, Mauricio Salcedo

**Affiliations:** 1 Laboratorio de Oncología Genómica, Unidad de Investigación Médica en Enfermedades Oncológicas, Hospital de Oncología, CMN Siglo XXI-IMSS, Mexico; 2 John Hopkins University, School of Medicine, Baltimore, MD, USA; 3 Unidad de Investigación Médica en Enfermedades Autoinmunes, Hospital de Especialidades, CMN Siglo XXI-IMSS México

## Abstract

**Background:**

Serial Analysis of Gene Expression (SAGE) is a new technique that allows a detailed and profound quantitative and qualitative knowledge of gene expression profile, without previous knowledge of sequence of analyzed genes. We carried out a modification of SAGE methodology (microSAGE), useful for the analysis of limited quantities of tissue samples, on normal human cervical tissue obtained from a donor without histopathological lesions. Cervical epithelium is constituted mainly by cervical keratinocytes which are the targets of human papilloma virus (HPV), where persistent HPV infection of cervical epithelium is associated with an increase risk for developing cervical carcinomas (CC).

**Results:**

We report here a transcriptome analysis of cervical tissue by SAGE, derived from 30,418 sequenced tags that provide a wealth of information about the gene products involved in normal cervical epithelium physiology, as well as genes not previously found in uterine cervix tissue involved in the process of epidermal differentiation.

**Conclusion:**

This first comprehensive and profound analysis of uterine cervix transcriptome, should be useful for the identification of genes involved in normal cervix uterine function, and candidate genes associated with cervical carcinoma.

## Background

One of the most frequent malignancies in women worldwide is the Uterine Cervical Carcinoma (CC), both in incidence and mortality and the first cause of death among the Mexican female population [1]. High-risk human papillomavirus (HPV) persistent infection is considered the most important risk factor associated with the development of this tumor [2,3]. Although HPV is a mandatory cause for CC, it is not sufficient to trigger all the changes required for its development [4].

A number of recent studies about gene expression profiles in *in vitro *HPV-infected cultured keratinocytes and from (CC) clinical samples have provided an initial notion of the changes in gene expression induced by HPV and in early CC [5-10]. Moreover, some studies have compared normal versus tumor-induced gene expression in cervical samples with the aim to identify potential tumor markers of clinical value [11-13].

At present, there are reports of genes expressed by keratinocytes derived from a normal human epidermis and from mouse uterus carried out by Serial Analysis of Gene Expression (SAGE) [14-17]. However, no such study exists for human cervix. Therefore, the aim of our study was to describe the first compendium of expressed genes in normal cervical epithelium, which is composed mainly by keratinocytes strongly influenced by hormones. To achieve this we used SAGE, which is capable of producing an accurate molecular picture of cervical tissue based on expressed genes, as the main methodology. As SAGE is not dependent on preexisting databases of expressed genes, it provides an unbiased view of gene expression profiles within the mRNA populations [18]. SAGE allows the simultaneous quantitative and qualitative analysis of thousands of gene transcripts based on two principles: first, 14 mers are sufficient to uniquely identify 95% of cell transcripts [19]; and second, cloning of these 14 bp tags serially with the insertion of a restriction enzyme recognition sequence as an anchor, the throughput is considerably increased. To obtain a catalog of expressed genes and their relative frequencies we performed database analysis to relate each tag to its corresponding gene [20]. As an important drawback of SAGE is that a large amount of messenger RNA (2.5–5 μg polyA RNA) is required, and our tissue supply was limited (a punch biopsy) we employed the MicroSAGE protocol in RNA thereof [21]. The present report describes a partial transcriptome of a sample derived from normal cervical epithelium used to construct a SAGE library with 30,418 sequenced tags.

## Results and discussion

### SAGE library derived from one normal uterine ectocervical sample

Our Sage library was obtained from ectocervical tissue from a 38 year old healthy woman with active sexual life, not taking any hormonal therapy, nor any other drug that could potentially alter cervical physiology, we designated this as SAGE_cervix_normal_B_1. Histological analysis of this sample by H&E revealed normal ectocervical tissue, approximately 80% epithelium and 20% stroma without evidence of glands. There were minimal inflammatory infiltrates in the periphery of the sample, considered normal for this type of tissue.

The SAGE library yielded 30,418 sequenced tags, which was used to generate a table, which represents genes expressed in normal human cervix. For a complete list of the expressed genes, please visit the SAGEmap website, [22,23]. The derived catalog of expressed genes represents the first attempt to generate a comprehensive and profound analysis of the cervical epithelium expression profile. The wealth of information obtained allows detection of genes involved in normal epithelium physiology, as well as possible target genes of HPV infection. In general, tag frequency in a typical SAGE experiment follows a normal distribution [24,25]. Table 1 summarizes the general statistics of this library. As seen there is a normal distribution, where only a limited number of tags were either highly expressed or at an extremely low frequency (4.4 and 4.9%, respectively). Tags with a frequency of 1 were not considered for quantitative purposes, because these are likely to represent artifacts of sequencing or of the SAGE procedure [26].

**Table 1:** SAGE data general statistics.

Frequency distribution ^*a*^	Tags	Individual Genes^*b*^
>200 tags	1,348 (4.4%)	5 (0.11%)

100–200 tags	2,371 (7.8%)^*c*^	21 (0.5%)^*d*^
20–100 tags	5,536 (18.19%)	80 (1.8%)
5–19 tags	6,349 (20.8%)	575 (13.2%)
2 – 4 tags	13,316 (43.7%)	3,050 (70.4%)
1 tag	1,498 (4.9%)	609 (14.1%)

Total	30,418 (100%)	4,340 (100%)

No of unique tags	8,062	
Tags with SAGE database matches	5,255	
No. of different transcripts matched	4,340	
No of poorly characterized transcripts	1,215	
Genes with known function^*e*^	2,453	

^*a*^Calculation of the frequency distributon of a given tag was based on total tags sequenced in library.

^*b*^Some genes have more than one tag, hence we search for individual genes in a database created in SQL language.

^*c*,*d*^Percentage of tags or genes in frequency group.

^*e*^based on FatiGO Datamining website [31]

### Representativity of the data

**Figure 1:**
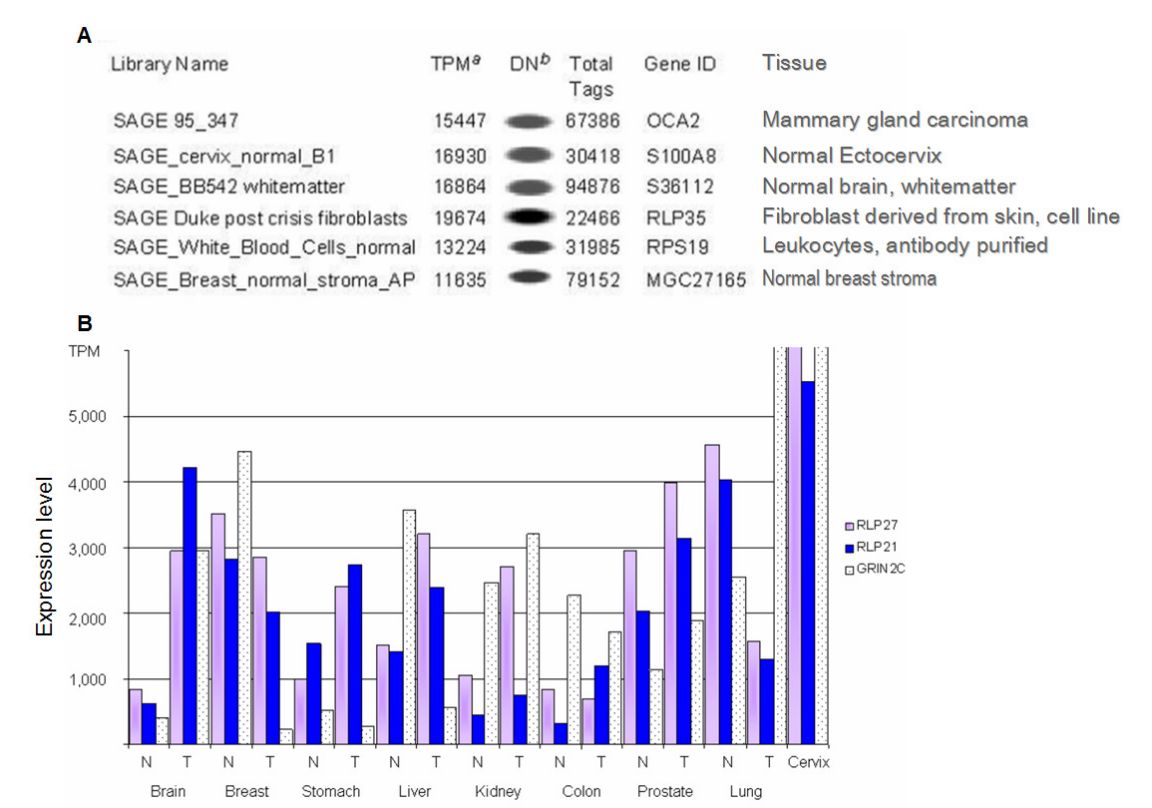
**A) Comparison of most expressed tags among different SAGE libraries**. Normalized expression levels (TPM) are similar between libraries with different total sequenced tags, indicating comparable messenger abundance among top expressed genes. Expression levels were obtained from SAGEmap website . ^*a*^TPM: Tags per million. Normalization to compare libraries with different numbers of sequenced tags. TPM is obtained by the following formula [(Tag frequency)(1000,000)/Total No. of sequenced tags]. ^*b*^DN: digital northern, indicating gene expression level for a specific gene in a library. **B. Graphical representation of expression levels (TPM) for three constitutive genes in several normal (N) and tumoral (T) tissues**. Brain tissue libraries: SAGE BB542 whitematter (N) and SAGE Brain medulloblastoma B 98 04 P117 (T). Breast: SAGE Breast normal organoid (N) and B SAGE Breast carcinoma epithelium AP DCIS6 (T). Gastric: SAGE normal gastric body epithelial (N) and SAGE Hiroshima GC W246T (T). Liver: SAGE normal liver (N) and SAGE Liver cholangiocarcinoma B K2D (T). Kidney: SAGE Duke Kidney (N) and SAGE_Kidney_carcinoma_B_D2 (T). Colon: SAGE NC2 (N) and SAGE Tu98 (T). Prostate: SAGE PR317 normal prostate (N) SAGE PR317 prostate tumor (T). Lung: SAGE normal lung (N) and SAGE Lung adenocarcinoma MD L10 (T). Expression levels are indicated as tags per million.

According to Zhang et al. [27], a study of SAGE data mining analysis of 300,000 tags, 75% of mRNA consists of transcripts expressed at more than five copies per cell, and, in general, transcripts are expressed at a range from one to 5,300 copies per cell. With this in mind, our ~30,000-tag library, represents 10% of the total tags analyzed by Velculescu et al. The most frequently represented tag in the current report had a frequency of 515 (16,930 tags per million TPM). An estimate of such data indicates that this gene tag has an expression level of ~5,150 copies per cell, similar to what is observed in digital northerns of other top expressed tags in SAGE libraries (Figure 1A). We have to keep in mind, however, that in certain tissues, some genes are expressed at much higher levels, such as growth hormone, with 149,630 TPM in pituitary gland [28]. Because SAGE analysis represents a qualitative and quantitative assay of messenger RNA abundance not biased by cloning or polymerase chain reaction efficiency [29], our data provide an estimate of the genes normally expressed by normal uterine cervix.

Among the most frequently expressed tags in our library (Table 2), some corresponded to ubiquitously expressed transcripts (*GRIN2C*, *FTH1*, *GNS*, *RPLP2*, *RPL21*). The presence of this type of genes is a common result in SAGE experiments with an expected heterogeneity in their expression levels [14,15,17,19], indicating a possible role as housekeeping genes (Figure 1B). In a report Velculescu *et al*., by means of data base analysis of SAGE libraries, found that ~1,000 genes are present in all normal or tumor tissues analyzed with over five copies per cell [30]. Hence, this list of genes identified by data mining is termed minimal transcriptome (i. e., the set of genes expressed by every cell), which represents genes constitutively expressed. In supplementary information of Velculescu's work [30] a search for the minimal transcriptome in our library, indicates >95% of housekeeping genes (data not shown), further validating the cervical library.

**Table 2:** Top 20 expressed genes in normal cervical tissue.

Tag sequence	Tags	TPM^*a*^	UniGene ID	Gene	Cluster name	Biological Function^*b*^
TACCTGCAGA	515	16930	Hs.416073	*S100A8*	S100 calgranulin A	Regulation of cell cycle progression and differentiation.
TAGGTTGTCT	356	11703	Hs.374596	*TPT1*	Tumor protein, translationally-controlled 1	Unknown
TTTCCTGCTC	276	9073	Hs.139322	*SPRR3*	Small proline-rich protein 3	Cross-linked envelope protein of keratinocytes
GAGGGAGTTT	201	6607	Hs.523463	*RPL27A*	Ribosomal protein L27a	Component of the ribosomal 60S subunit
GTGACCACGG	188	6180	Hs.436980	*GRIN2C*	Glutamate receptor, N-methyl D-aspartate 2C	Ionotropic glutamate receptor
GTGGCCACGG	184	6049	Hs.112405	*S100A9*	S100 calcium binding protein A9 (calgranulin B)	Regulation of cell cycle progression and differentiation.
GGGCTGGGGT	173	5687	Hs.425125; Hs.90436	*RPL29; SPAG7*	Ribosomal protein L29; Sperm associated antigen 7	Component of the ribosomal 60S subunit.
GCATAATAGG	168	5523	Hs.381123	*RPL21*	Ribosomal protein L21	Component of the ribosomal 60S subunit.
TCAGATCTTT	161	5292	Hs.446628	*RPS4X*	Ribosomal protein S4, X-linked	Component of the ribosomal 40S subunit.
GTTGTGGTTA	155	5095	Hs.99785		FLJ21245	Unknown
GGATTTGGCC	151	4964	Hs.437594	*RPLP2*	Ribosomal protein, large P2	Component of the ribosomal 60S subunit.
TTGGGGTTTC	143	4701	Hs.448738	*FTH1*	Ferritin, heavy polypeptide 1	Important for iron homeostasis, stores iron in a soluble, nontoxic, readily available form.
TTGGTCCTCT	138	4536	Hs.381172	*RPL41*	Rribosomal protein L41	Component of the ribosomal 60S subunit.
TGCACGTTTT	130	4273	Hs.265174	*RPL32*	Ribosomal protein L32	Component of the ribosomal 60S subunit.
ACAAAGCATT	128	4208	Hs.369982	*IGFBP5*	Insulin-like growth factor binding protein 5	IGF-binding proteins prolong the half-life of the IGFs and have been shown to either inhibit or stimulate the growth promoting effects of the IGF on cell culture.
AGGGCTTCCA	122	4010	Hs.401929	*RPL10*	Ribosomal protein L10	Component of the ribosomal 60S subunit.
CCACTGCACT	114	3747	Hs.107003	*CCNB1IP1*	Cyclin B1 interacting protein 1	Functions in progression of the cell cycle through G(2)/M.
GGCAAGCCCC	107	3517	Hs.148340	*RPL10A*	ribosomal protein L10a	Component of the ribosomal 60S subunit.
CTGGGTTAAT	105	3451	Hs.334534	*GNS*	Glucosamine (N-acetyl)-6-sulfatase	Lysosomal enzyme found in all cells. It is involved in the catabolism of heparin, heparan sulphate, and keratan sulphate.
TGCACTTCAA	103	3386	Hs.62886	*SPARCL1*	SPARC-like 1 (mast9, hevin)	Calcium ion binding

^*a*^TPM: Tags per million; [(Tag frequency)(1000,000)/Total No of sequenced tags].

^*b*^Biological function obtained from SOURCE, at

### Spectrum of genes expressed by normal cervical tissue

**Figure 2:**
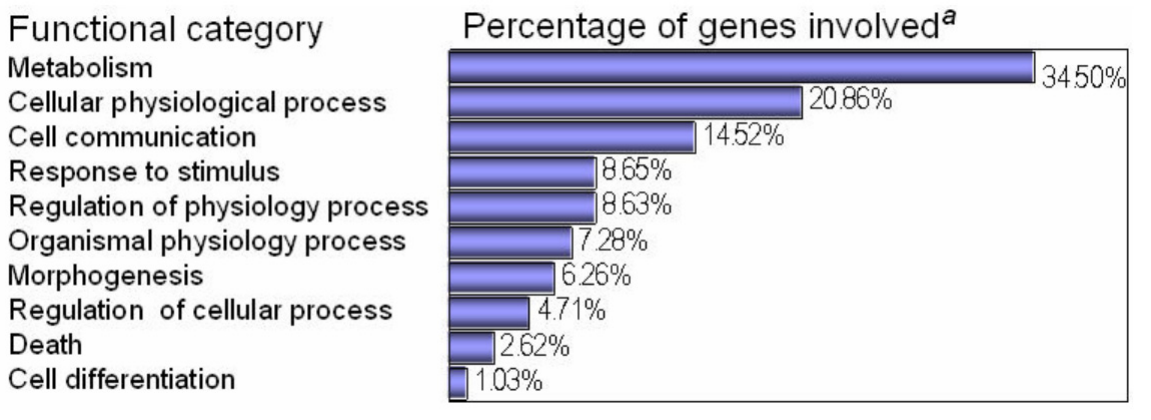
**Functional categories assigned to individual genes identified in normal cervical SAGE library**. Genes can be assigned in different functional categories. ^*a*^The percentage was calculated with 3,764 initial genes from which 2,720 genes had Gene Ontology classification.

To obtain better knowledge of the functional categories of global gene expression profile, we employed the Fatigo Data mining website [31,32]. Figure 2 shows the distribution of expressed genes by functional categories defined by the Gene Ontology Consortium. As seen, the most frequent individual transcripts correspond to genes involved in maintenance and basic metabolism. On the other hand, genes corresponding to other processes such as cell growth regulation, morphogenesis, cell differentiation, or death were not as frequently expressed.

### Top expressed non-ubiquitous genes in normal cervical tissue mainly correspond to epithelial growth and differentiation

It was important to distinguish which non-ubiquitous genes were predominantly expressed in normal cervix. As seen in table 2, genes related with epithelial differentiation and squamous architectural maintenance are abundantly represented in our library. These include *S100A8*, *S100A9*, and *SPRR3*, that belong to a complex of genes that are subject to coordinate regulation during keratinocyte differentiation. This complex has been called the epidermal differentiation complex (EDC) and is located on the 1q21 chromosome [33,34]. These genes share spatial and temporal expression and interrelated functions and are grouped in three related gene families: cornified envelope precursor proteins (involucrin, loricrin, and the small proline-rich proteins [SPRRs]); intermediate filament-associated proteins (profilaggrin and trichohyalin), and calcium binding proteins (the S100As) [reviewed in [35]]. Approximately 30 genes belonging to the EDC are clustered together in a 200 Mb region, from which there are 20 genes expressed the in cervical SAGE library (Table 3).

**Table 3:** Genes belonging to 1q21 epidermal differentiation complex (EDC) expressed in cervical tissue

TAG Sequence	TAGS	TPM^*a*^	UniGene ID	Gene ID	Gene name
TACCTGCAGA	515	16930	Hs.416073	*S100A8*	S100 calcium binding protein A8
TTTCCTGCTC	276	9073	Hs.139322	*SPRR3*	Small proline-rich protein 3
GTGGCCACGG	184	6049	Hs.112405	*S100A9*	S100 calcium binding protein A9
GATCAGGCCA	18	591	Hs.275243	*S100A6*	S100 calcium binding protein A12
GATCTCTTGG	17	558	Hs.38991	*S100A2*	S100 calcium binding protein A2
AGCAGATCAG	15	493	Hs.400250	*S100A10*	S100 calcium binding protein A10
CGTGGGACAC	12	394	Hs.110196	*NICE-1 (C1orf42)*	Chromosome 1 open reading frame 42
CAGGCCCCAC	12	394	Hs.417004	*S100A11*	S100 calcium binding protein A11
ATGTGTAACG	8	263	Hs.81256	*S100A4*	S100 calcium binding protein A4
GAGCAGCGCC	7	230	Hs.112408	*S100A7*	S100 calcium binding protein A7
ATGATCCCTG	7	230	Hs.355542	*SPRR2A*	Small proline-rich protein 2A
TTGTGATGTA	7	230	Hs.85844	*TPM3*	Tropomyosin 3
TTCCCTTACC	6	197	Hs.244349	*LCE3D*	Late cornified envelope 3D
GTCAGGGGAT	5	164	Hs. 12341	*ADAR1*	ADAR Adenosine deaminase, RNA-specific
CCCTTGAGGA	5	164	Hs.1076	*SPRR1B*	Small proline-rich protein 1B (cornifin)
CCCAGATGAT	4	131	Hs.7854	*SLC39A1*	Solute carrier family 39 (zinc transporter), member 1
AACCCTAAAA	2	65	Hs.75117	*ILF2*	Interleukin enhancer binding factor 2, 45 kDa
GCAAATTTGA	2	65	Hs.6396	*JTB*	Jumping translocation breakpoint
CAAGGATCTA	2	65	Hs.355906	*NICE-3 (C1orf43)*	Chromosome 1 open reading frame 43
CAAGGATCTA	2	65	Hs.490551	*UBAP2L (NICE-4)*	Ubiquitin associated protein 2-like
AGCCACTGCA	2	65	Hs.516439	*IVL*	Involucrin

^a^TPM: Tags per million.

### End point RT-PCR analysis confirms expression of genes detected by SAGE

**Figure 3:**
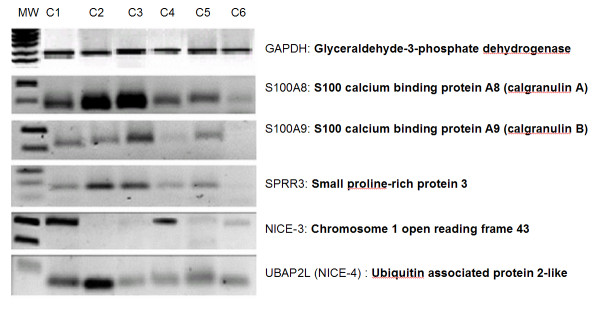
**Expression of genes clustered in 1q21, in normal cervical tissues**. One hundred nanograms of total RNA purified of each sample was used in one RT-PCR reaction with gene specific primers; then one tenth of each RT-PCR reaction was subjected to agarose gel electrophoresis. MW: molecular weight marker; C1–C6 six different normal cervical samples

It was important to confirm the expression of some EDC representative genes in different normal cervical tissues by a different technique. For this, we chose end point reverse transcriptase polymerase chain reaction (RT-PCR) analysis. Figure 3 shows the expression of five EDC genes in HPV negative tissue samples with no histopathologic lesion. As expected, the majority of cases expressed these genes. However, there were some differences in the level of expression among the different normal samples. This could be due to the fact that samples were taken on different days of the menstrual cycle (hormonal influence) or to unknown physiological differences among biological systems.

### Minor expression of fibroblast-related genes in cervical tissue

The gene expression catalog reported on here was obtained from a heterogeneous population of cells composed mainly of epithelial keratinocytes in dissimilar differentiation stages (*basale*, *spinosum *and *granulosum strata*). Nevertheless, these tissues also contain fibroblasts associated with connective, besides other minor cell populations. To know which genes are related to fibroblasts, we compared a SAGE library derived from neonatal foreskin primary fibroblasts (Agnes Baross, British Columbia Genome Sciences Centre). We found 923 gene tags shared by both libraries, which could due to the presence of fibroblasts in the Cervix SAGE library (supplementary information). Shared genes with known biological function reveal that processes as signal transduction, regulation of transcription and cell adhesion are mainly involved. We consider important to identify minor contributions to global gene expression profile in a heterogeneous cell population; however, it is important to note that unknown differences between cervical and neonatal foreskin fibroblasts could exist.

## Conclusion

To our knowledge, this is the first effort to achieve a global profile of gene expression in normal cervical tissue. This was accomplished by means of a methodology that produced an accurate catalog of expressed genes in this tissue. Analysis of gene expression revealed genes involved in keratinocyte differentiation. These genes have not been detected in cervical epithelium by traditional methodologies such as RT-PCR or *in situ *hybridization. Although our SAGE library was derived from a single donor, the majority of samples analyzed expressed the genes selected, indicating reproducibility in human samples. SAGE methodology is a complex and expensive analysis mainly due to the great sequencing efforts required to achieve SAGE libraries. Nevertheless, the overwhelming information derived from these justifies the effort and provides better knowledge of cervical biology and physiology. In a near future, it could also provide an insight of cervical physiology or HPV infection and in other pathologies affecting cervical tissue.

## Methods

### Tissues

Normal cervices were obtained from women with negative Pap smears, confirmed by histopathological analysis, attending at the Dysplasia Clinic at General Hospital of Mexico, SS who had been subjected to hysterectomy due to uterine myomatosis. All patients were in reproductive age and none of them received hormonal therapy or contraceptives. All the described procedures were evaluated and approved by the local ethics committee of the Mexican Institute of Social Security. Written informed consent was obtained from all the patients. All tissue samples were longitudinally divided in three sections, the central part was snapped frozen in liquid nitrogen and stored at -70°C until nucleic acid extraction, and the other two were fixed overnight in 70% ethanol and were paraffin embedded at the Department of Pathology, Oncology Hospital, National Medical Center SXXI, Mexico. Serial sections from these fractions stained by Haematoxilin/ Eosin were inspected for representativity of the tissue.

### HPV detection and typing

Genomic DNA was extracted from the phenol phase left by the TRIzol reagent (Gibco BRL, USA) RNA isolation protocol and amplified by PCR with MY11/MY09 primers [36] (Table 4). PCR products were separated by electrophoresis on 1% agarose gel. Only HPV negative samples were included in this study.

**Table 4:** Oligonucleotides sequences used in this work.

Gene	Sense (5'→3')	Antisense (5'→3')	Annealing temperature (°C)^*a*^	Product size (bp)	Reference
HPV*	GCMCAGGGWCATAAYAATGG	CGTCCMARRGGAWACTGATC	55	450	[36]
S100 A8	ATGCCGTCTACAGGGATGAC	ACGCCCATCTTTATCACCAG	58	160	This paper
S100 A9	TCAGCTGGAACGCAACATAGA	TCAGCTGCTTGTCTGCATT	56	205	This Paper
SPRR3	TTCCACAACCTGGAAACACA	TTCAGGGACCTTGGTGTAGC	55	174	This paper
NICE-3	ACGGCTATGAAACAGCCCGCTA	GCACATTGCAACTGACTGGCTT	57	330	This paper
NICE-4	ACGGAATCCAATGAGGAAGGCA	TCAGTATTGGCTGGCTCTGCAT	57	294	This paper
GAPDH	CATCTCTGCCCCCTCTGCTGA	GGATGACCTTGCCCACAGCCT	60	205	[38]

^*a*^Tm was calculated using primerquest program from [39]; however, it was necessary to adjust Tm in some cases.

### Micro SAGE protocol

Micro SAGE was performed according to Datson *et al*. [21] with minor modifications, by means of the Invitrogen's I-SAGE kit (Invitrogen, San Diego, CA USA). RNA isolation was done in TRIzol according to manufacturer's instructions. Five μg of total RNA was used as input material. A heating step was introduced at 65°C for 10 minutes followed by 2 minutes on ice to allow a better separation of concatenamers [37]. Products greater than 300 bp and smaller than 2,000 bp were excised, extracted and cloned in the *Sph*I site of pZero vector. Clones were selected and screened for inserts by PCR. Cervix library was sequenced by Agencourt through SAGE sequencing service (CGAP collaboration, GR). Sequence files were analyzed with the SAGE300 software [18,20], which identifies the anchoring enzyme sites and extracts the two tags flanked by *Nla*III site. Gene identity and UniGene cluster assignment of each SAGE tag was obtained by using the tag-to-gene "reliable" map, from SAGEmap NCBI site [22,23]. The tags extracted were uploaded to SAGEmap and corresponding accession numbers were retrieved using the *H. sapiens *NCBI-GenBank database.

### Reverse Transcription-Polymerase Chain Reaction (RT-PCR) analysis

Total RNA was extracted from six normal cervical tissues using TRIzol, quantified by densitometric analysis and its quality evaluated by denaturing gel electrophoresis. Contaminiating DNA was digested and removed with Rnase-free Dnase (Promega). Expression analysis was performed using 100 ng total RNA in a RT-PCR reaction (Access RT-PCR System, Promega). The mRNA was reverse-transcribed at 48°C for 45 min. After an initial denaturation at 94°C for 2 minutes, the double stranded cDNA synthesized was amplified for 40 cycles with denaturation at 94°C for 30 seconds, annealing at 54–60°C for 1 minute and extension at 70°C for 2 minutes with specific oligonucleotides (Table 4) in a Perkin Elmer 480 Thermocycler.

Sense and antisense sequence of oligonucleotides for S100 A8 and 9, SPRR3, NICE-3 and -4 genes were designed with the program Primerquest [38]. GAPDH gene expression was used as an internal control.

## Competing interests

The author(s) declare that they have no competing interests.

## Authors' contributions

C.P.P. carried out the microSAGE protocol, real time RT-PCR validations, the bioinformatics analysis and writing the manuscript. G.R. provided sequencing of SAGE library. J.M. helped to write the manuscript and participated in discussions. H.G. A.H. and P.P.S. helped for the bioinformatics analysis and database comparisons. M.S. is the principal investigator and was involved in the conceptualization, design and writing of the manuscript. All authors read and approved the final manuscript.

## Supplementary Material

Additional data file 1 Tags shared between fibroblast and cervix. Tags founded in SAGE_cervix_normal_B_1(30418 tags) BJ dermal fibroblasts (57573 tags) and SAGE_cervix_normal_B_1 (30418 tags). Ubiquous tags were deleted in both libraries. BJ dermal fibroblast library was derived from neonatal foreskin primary fibroblasts cultured in Ham's F10 medium supplemented with 10% fetal bovine serum, 100 U/ml penicillin, and 100 ug/ml streptomycin. Library was developed by Agnes Baross at British Columbia Genome Sciences Centre. Columns are: Gene TAG, Cluster ID and Gene Name Click here for file
